# Preparation of a Z-Type g-C_3_N_4_/(A-R)TiO_2_ Composite Catalyst and Its Mechanism for Degradation of Gaseous and Liquid Ammonia

**DOI:** 10.3390/ijms232113131

**Published:** 2022-10-28

**Authors:** Jiaming Zhu, Zuohua Liu, Hao Wang, Yue Jian, Dingbiao Long, Shihua Pu

**Affiliations:** 1 Chongqing Academy of Animal Sciences, Chongqing 402460, China; 2 Scientific Observation and Experiment Station of Livestock Equipment Engineering in Southwest, Ministry of Agriculture, Chongqing 402460, China

**Keywords:** photocatalysis, ammonia, degradation, mechanism

## Abstract

In this study, an (A-R)TiO_2_ catalyst (ART) was prepared via the sol–gel method, and g-C_3_N_4_ (CN) was used as an amendment to prepare the g-C_3_N_4_/(A-R)TiO_2_ composite catalyst (ARTCN). X-ray diffraction (XRD), scanning electron microscopy (SEM), transmission electron microscopy (TEM), Raman spectroscopy, N_2_ adsorption–desorption curves (BET), UV–Vis diffuse absorption spectroscopy (UV–Vis DRS), and fluorescence spectroscopy (PL) were used to evaluate the structure, morphology, specific surface area, optical properties, and photocarrier separation ability of the catalysts. The results showed that when the modifier CN content was 0.5 g, the dispersion of the ARTCN composite catalyst was better, with stronger light absorption performance, and the forbidden band width was smaller. Moreover, the photogenerated electrons in the conduction band of ART transferred to the valence band of CN and combined with the holes in the valence band of CN, forming Z-type heterostructures that significantly improved the efficiency of the photogenerated electron-hole migration and separation, thus increasing the reaction rate. Gaseous and liquid ammonia were used as the target pollutants to investigate the activity of the prepared catalysts, and the results showed that the air wetness and initial concentration of ammonia had a great influence on the degradation of gaseous ammonia. When the initial concentration of ammonia was 50 mg/m^3^ and the flow rate of the moist air was 0.9 mL/min, the degradation rate of gaseous ammonia by ARTCN-0.5 reached 88.86%, and it had good repeatability. When the catalytic dose was 50 mg and the initial concentration of NH_4_^+^ was 100 mg/L, the degradation rate of liquid ammonia by ARTCN-0.5 was 71.60% after 3 h of reaction, and small amounts of NO_3_^−^ and NO_2_^−^ were generated. The superoxide anion radical (·O_2_^−^) and hydroxyl radical (·OH) were the main active components in the photocatalytic reaction process.

## 1. Introduction

Ammonia is a colorless alkaline gas that causes strong irritation, mainly from agricultural fertilization, animal husbandry, and the use of antifreeze [1]. Estimates have shown that China emits 10 to 15 million tons of ammonia into the air every year, almost double the total of the United States and the European Union [2]. Large-scale ammonia emissions will not only cause diseases in animals and humans, such as central muscle paralysis and bronchitis, but also cause global climate change [3]. Ammonia reacts with oxides in the air, producing particulate matter such as ammonium sulfate and ammonium nitrate, which are the main factors that form haze [4]; thus, controlling ammonia emissions is beneficial to controlling haze [5]. In addition, ammonia gas will return to the surface through atmospheric dry and wet deposition, leading to the eutrophication of water and affecting the stability of the ecosystem [6]. Traditional ammonia treatment processes have high costs and poor stability, which may aggravate the secondary pollution of the environment [7].

In 1972, Fujishima discovered that TiO_2_ could photolyze water under ultraviolet light, and this photocatalytic reaction has been widely used in environmental governance, energy development, biological applications, self-cleaning materials, antibacterial applications, sensors, and other fields due to its thorough reaction and lack of secondary pollution [8]. For example, A. Enesca et al. [9] prepared doped tin oxide films with different dopant concentrations through spray pyrolysis deposition and found that the photodegradation efficiency of the SnO_2_ film could reach about 30% under the condition of zinc doping. X. Hu et al. [10] introduced and discussed the current challenges and future development prospects of CO_2_ photoreduction for hydrocarbon fuels. X. Liu et al. [11] prepared CdS@ZIS-SV, and its hydrogen production rate reached 18.06 mmol/g/h, which was 16.9 and 19.6 times that of original CdS (1.16 mmol/g/h) and ZIS (0.92 mmol/g/h) materials, respectively. In ammonia gas degradation, P.A. Kolinko et al. [12] and H.M. Wu et al. [13] pointed out that the N element had various valence states. Herein, its main product was N_2_, and its by-products were N_2_O, NO_2_^−^, and NO_3_^−^. Among the many photocatalytic materials, TiO_2_ has become photocatalyst with the most potential due to its advantages such as good chemical stability, safety, non-toxicity, low cost, and strong REDOX ability [14]. Most studies have indicated that the degradation performance of anatase (A-TiO_2_) is better than rutile (R-TiO_2_), and reports on TiO_2_ have mainly used the anatase phase [15]. Shen et al. [16] stated that when the A-TiO_2_ and R-TiO_2_ phases formed a heterogeneous structure, an internal electric field could form, thus promoting the transfer of charges on the interface and improving photocatalytic activity. Das et al. [17] prepared a mixed anatase/rutile crystal structure that had a higher photocatalytic performance than commercial P25 under visible light. Xiong et al. [18] showed that mixed crystalline anatase and rutile TiO_2_ nanoparticles exhibited a high photocatalytic carbon dioxide reduction capacity. However, its high bandgap width (3.2 eV for anatase and 3.0 eV for rutile) resulted in a response to only high-energy UV light, and its photogenerated charge carriers were easy to recombine, thus limiting its catalytic activity [19]. For example, Guarino et al. [20] sprayed TiO_2_ on a wall with a total area of 150 m^2^ at a spray quantity of 70 g/m^2^ and using a 36 W UV light as the light source, and the degradation rate of ammonia was found to be about 30%.

Photocatalytic materials can be used to form heterogeneous structures. For example, Shihua Pu et al. [21] achieved the degradation of ammonia under sunlight for the first time through the Cu_2_O improvement of {001}TiO_2_, and the degradation rate of ammonia was found to be more than 80% within 2 h. However, due to the photocorrosion of Cu_2_O itself, the degradation rate of ammonia gas was only maintained at 40% after four repeated uses, making the choice of amendment very important. g-C_3_N_4_ has shown a narrow band gap, with a wide range of light responses and good thermal stability, chemical stability, and strong corrosion resistance [22]. Most studies have found that the formation of heterostructures through the preparation of g-C_3_N_4_/TiO_2_ composite catalysts could broaden the solar spectral response of the catalyst, and photogenerated charge carriers are not easy to recombine [23]. For example, Li et al. [24] constructed a g-C_3_N_4_/TiO_2_ heterostructure that achieved effective photoinduced electron-hole separation in the photocatalytic process and showed a good photocatalytic effect and cyclic stability. Zhao S et al. [25] used synthesized g-C_3_N_4_/TiO_2_ to degrade phenol with 2.41 and 3.12 times g-C_3_N_4_ and TiO_2_ contents, respectively. Sun et al. [26] used synthetic g-C_3_N_4_/TiO_2_ to degrade methylene blue with 1.85 and 4 times pure g-C_3_N_4_ and TiO_2_ content, respectively; this showed that it was feasible to improve TiO_2_ degradation capacity by using g-C_3_N_4_ as an amendment, but a study on ammonia degradation has not been previously reported.

In this study, an (A-R)TiO_2_ catalyst (ART) was prepared via the sol–gel method, where g-C_3_N_4_ (CN) was used as an amendment to prepare g-C_3_N_4_/(A-R)TiO_2_ (ARTCN) with a Z-type heterostructure, which improved the efficiency of photogenerated electron-hole migration and separation. In this study, using gaseous ammonia and ammonia as the target pollutants, we explored the ARTCN degradation of ammonia, the mechanism of performance improvement, the intermediate, and the reaction process. We also studied the repeated use of ARTCN, as evidence on the properties and mechanism noted in this research regarding the effective governance of ammonia pollution has been relatively scarce, and this research could provide a certain theoretical basis for the management of ammonia.

## 2. Results

### 2.1. Analysis of the Characterization Results

#### 2.1.1. XRD Analysis

When 2θ = 25.28°, 36.95°, 37.80°, 38.55°, 48.04°, 53.89°, 55.06°, 62.68°, 70.31°, 75.03°, and 76.02°, the characteristic peaks corresponding to the (101), (103), (004), (112), (200), (105), (211), (204), (220), (215), and (301) crystal planes, respectively, were almost consistent with the anatase TiO_2_ (JCPDS No. 21-1272) standard cards [27]. Furthermore, 2θ = 27.45°, 36.09°, 39.19°, 41.26°, 44.05°, 54.32°, 56.64°, 64.04°, and 69.01° corresponded to the (110), (101), (200), (111), (210), (211), (220), (310), and (301) crystal planes, respectively, which was almost consistent with the rutile TiO_2_ (JCPDS No. 21-1276) standard card [28]. The characteristic peaks at 2θ = 13.1°and 27.5° belonged to the (100) and (002) planes, respectively which was largely in line with g-C_3_N_4_ (JCPDS87-1526) [29].

**Figure 1:**
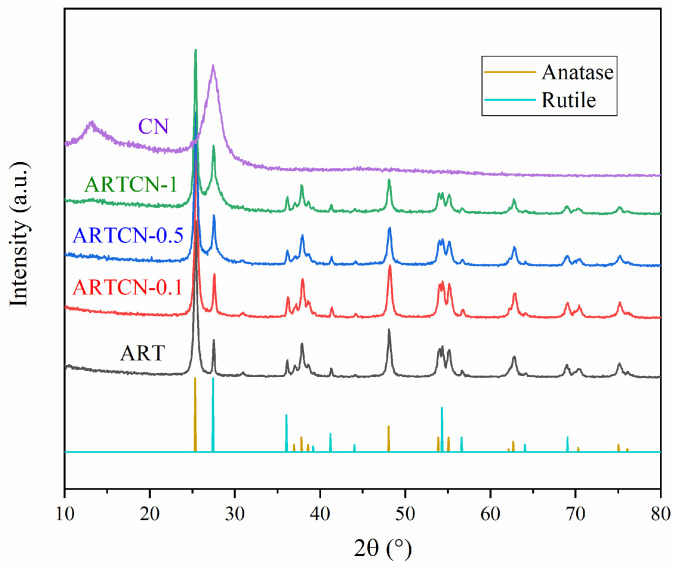
XRD patterns of the samples.

Figure 1 shows the XRD patterns of the prepared samples. We found that ART contained not only the characteristic peaks of the anatase phase but also the characteristic peaks of the rutile phase, indicating that it was a mixed crystal type, which was the prepared (A-R)TiO_2_ catalyst. CN had a weak diffraction peak near 13.1°, corresponding to the (100) crystal plane of CN, which was formed by the 3-S monotriazine structural unit of the plane [30]. There was a strong diffraction peak at 27.50°, which was caused by the layered accumulation of graphite in the conjugate plane. The diffraction peak of the ARTCN-X composite corresponded to pure ART, which indicated that CN did not enter the ART lattice and was only attached to its surface. There was no diffraction peak in the composite samples of ART and CN at 13.1°, because the amount of CN was small and had a weak peak. Although the peaks of CN at 27.50° and R-TiO_2_ at 27.45° coincided, we observed that as the composite ratio increased from 0.1 to 1, the composite gradually widened around 27°, indicating that the composite material was successfully prepared.

The accuracy was further verified by calculating the rutile content of several materials [31], and the calculation results are shown in Table 1. We found that the rutile contents of ART, ARTCN-0.1, ARTCN-0.5, and ARTCN-1 were 23.49%, 25.06%, 29.43%, and 34.61%, respectively. High temperatures were conducive to the conversion of A-TiO_2_ into R-TiO_2_, while ART, ARTCN-0.1, ARTCN-0.5, and ARTCN-1 were prepared at the same temperature; thus, the increased rutile content was not rutile in the real sense but rather the added amendment of CN.

Table 1 shows the rutile content of each catalyst. The formula (Equation (1)) is given as follows:

(1) $$X_{R}=\frac{1}{1+0.8\times \frac{I_{A}}{I_{R}}}$$

where X_R_ is the rutile content, I_A_ is the peak intensity of anatase at 2θ = 25.28°, and I_R_ is the peak intensity of rutile at 2θ = 27.24°.

#### 2.1.2. Raman Analysis

**Figure 2:**
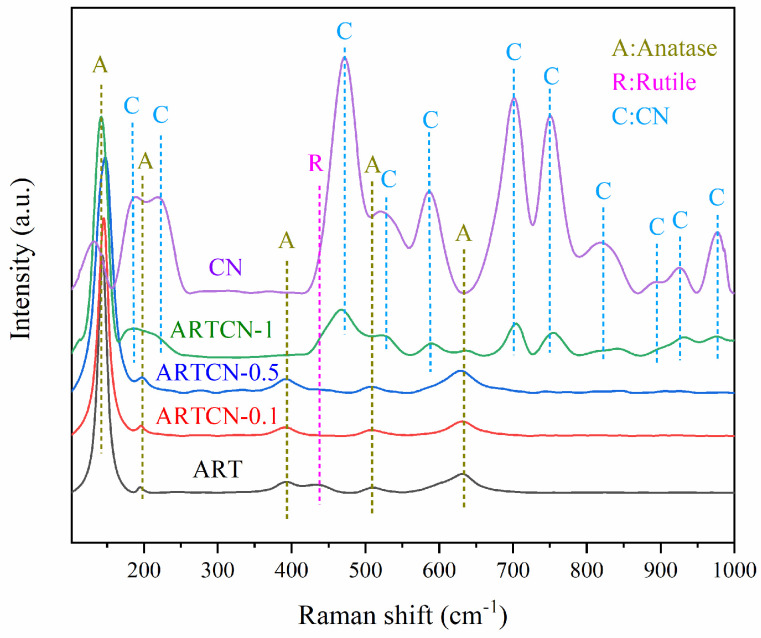
Raman spectra of the samples.

The crystal form and structure of the catalyst were further determined by Raman spectroscopy, and Figure 2 shows the Raman spectra of the prepared catalyst. The anatase phase of TiO_2_ corresponded to ν = 144 cm^−1^, ν = 197 cm^−1^, ν = 392 cm^−1^, ν = 514 cm^−1^, and ν = 635 cm^−1^ [32], while ν = 437 cm^−1^ corresponded to rutile TiO_2_. The reason why other characteristic peaks did not appear was that the content of rutile in ART was low (as indicated by the peak intensity of XRD). ART and composite ARTCN-X both had anatase and rutile peaks, especially the CN peak of ARTCN-1, which further validated the XRD results.

#### 2.1.3. Morphology and Lattice Spacing Analysis

**Figure 3:**
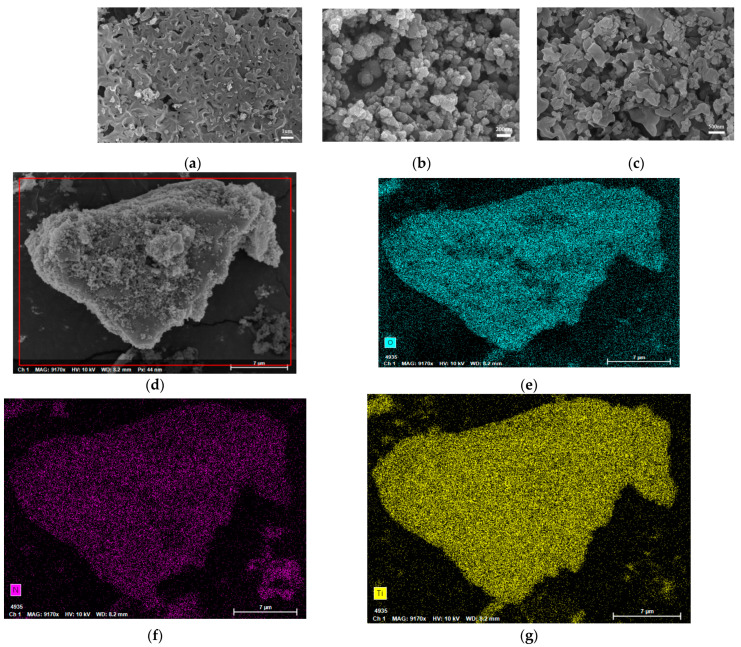

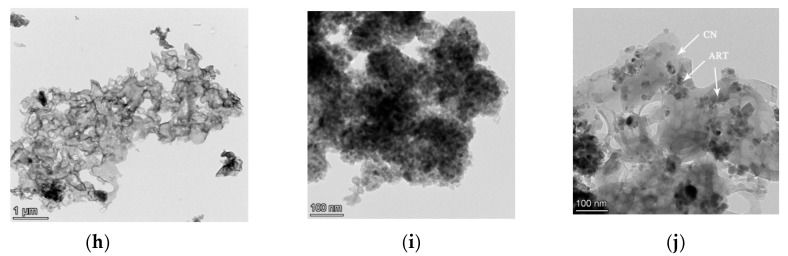
SEM images of CN (**a**), ART (**b**), and ARTCN-0.5 (**c**); images and corresponding EDS elemental mapping images (**d**–**g**) of the ARTCN-0.5, TEM of CN (**h**), ART (**i**), and ARTCN-0.5 (**j**).

According to Figure 3a,h, CN was a curved and folded film with certain holes. As shown in Figure 3b,i, ART consisted of a particle with a uniform size but serious agglomeration, and Figure 3c shows that CN in ARTCN-0.5 was no longer a whole film but fragmented into many small pieces; thus, its position relative to ART could not be clearly observed. Combined with Figure 3d–g, we clearly observed that elements O, Ti, and N were evenly distributed in ARTCN-0.5. This showed that a composite material with a heterogeneous structure was synthesized. In addition, as clearly shown in Figure 3j, the ART agglomeration phenomenon was significantly improved after the improvement of CN, which indicated that the utilization of light by ART in the composite ARTCN-0.5 was further enhanced.

#### 2.1.4. Analysis of Adsorption–Desorption of N_2_

**Figure 4:**
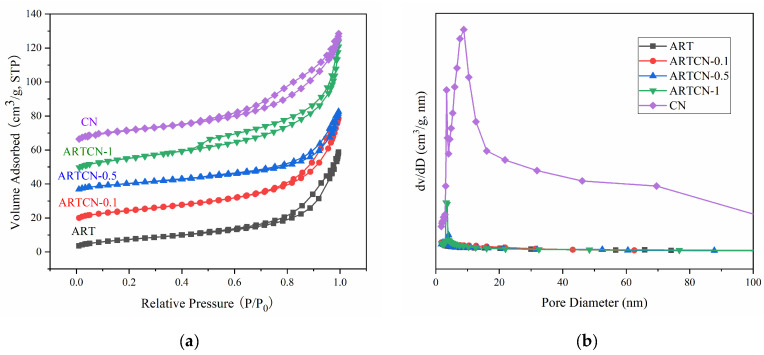
N_2_ adsorption–desorption curve (**a**) and pore size distribution (**b**) of the samples.

The Brunauer–Emmett–Teller (BET) method was used to calculate the surface area of the catalyst, and the Barrett–Joyner–Halenda (BJH) method was used to analyze the pore size and pore volume. The catalysts shown in Figure 4a all exhibited typical nitrogen adsorption–desorption isotherms of type IV, and the hysteresis loops of the catalysts all showed obvious openings, indicating the formation of mesoporous catalysts [33]. Figure 4b shows the pore size distribution of the catalyst, which showed that the pore size of the prepared catalyst was mainly concentrated at 0–20 nm, indicating that the particle size distribution of the catalyst was narrow. Combined with the data in Table 2, we found that the pores of the catalysts showed little difference, while the specific surface area and pore diameter of the catalysts increased with increasing CN content.

#### 2.1.5. Optical Performance Analysis

**Figure 5:**
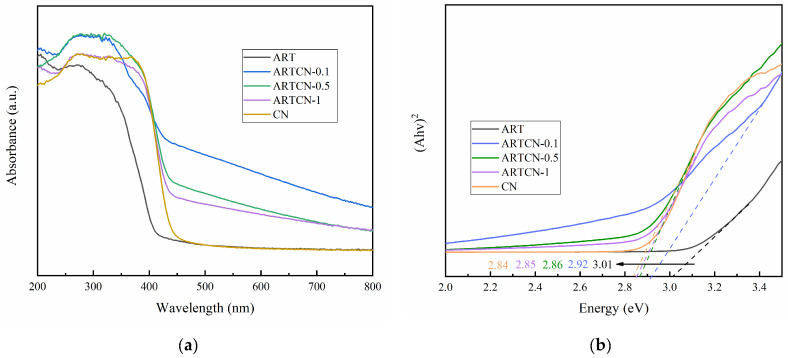
Optical properties (**a**) and the band gap width (**b**) of the samples.

The optical absorption properties of the prepared samples were investigated via the UV–Vis absorption spectra, and the results are shown in Figure 5a. The optical absorption intensity of the ARTCN-X composite material improved by CN widened in the visible light range because the specific ART surface area improved as CN increased, and the agglomeration phenomenon also significantly improved. These results indicated that CN could effectively expand the optical absorption range of ART, thus improving the response and utilization efficiency of visible light. In addition, the band gap width of the prepared material was calculated with the Kubelka-Munk method [34], as shown in Figure 5b. The bandgap widths of ART, ARTCN-0.1, ARTCN-0.5, ARTCN-1, and CN were 3.01, 2.92, 2.86, 2.85, and 2.84 eV, respectively. First, we clearly observed that the band gap width of ART was smaller than the 3.12 eV value reported in the literature, because heterostructures would form between the anatase phase TiO_2_ and rutile phase TiO_2_, enhancing the ability of photogenerated carrier separation [35]. Secondly, we found that with the increase in the CN content of the amendment, the band gap width of the composite ARTCN-X became significantly smaller than that of ART and approached that of CN, which was consistent with other studies [36].

#### 2.1.6. PL and EPR Analysis

**Figure 6:**
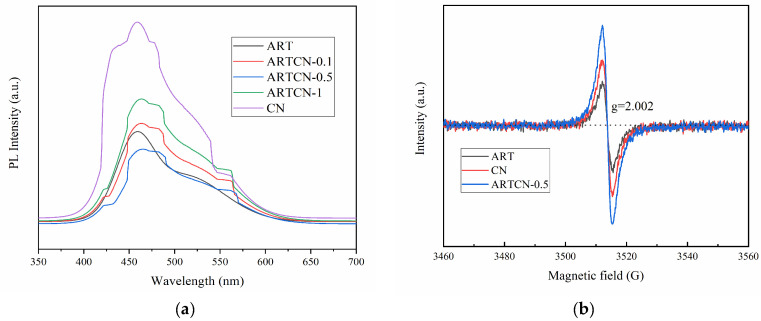
PL (**a**) and (**b**) EPR of the samples.

A PL spectrum can be used to study the separation of the photogenerated carriers in semiconductors, where the lower the peak intensity, the lower the recombination rate of the photogenerated carriers and the higher the photocatalytic activity [37]. As shown in Figure 6a, ARTCN-0.5 had the lowest peak intensity, indicating that its photocatalytic activity was the highest, which further indicated that as an amendment, the amount of CN addition did not follow the logic of “the more content the better” but had an appropriate ratio with ART. In addition, we observed that the wide wavelength range of ARTCN-X after CN modification in the interval of 451.8–468.8 nm was attributed to the oxygen vacancy that contained two captured electrons, which both promoted the formation of superoxide radicals (·O_2_^−^) and hydroxyl radicals (·OH) and was favorable for photocatalytic degradation.

Additional EPR spectra that were collected at room temperature provided information regarding the oxygen vacancies (Ov). As shown in Figure 6b, the signal at g = 2.002 corresponded to Ov [38]. We found that all samples had oxygen vacancy signals, and the intensity of ARTCN-0.5 was stronger than that of single ART and CN, which indicated that there was a large amount of Ov that could effectively inhibit the recombination of electrons and holes and improve the photocatalytic activity, thus confirming the results shown in Figure 6a.

### 2.2. Photocatalytic Performance Test Results

#### 2.2.1. Study on the Photocatalytic Degradation of Gaseous Ammonia

**Figure 7:**
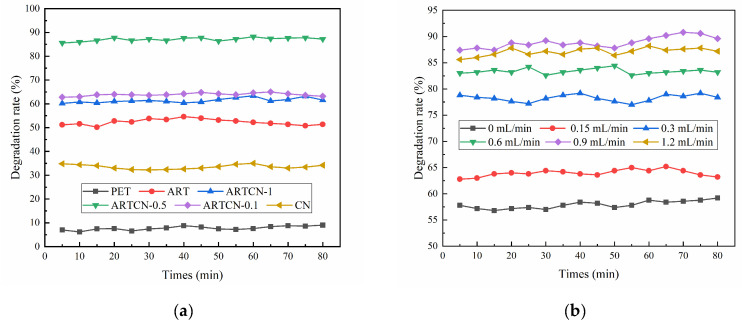

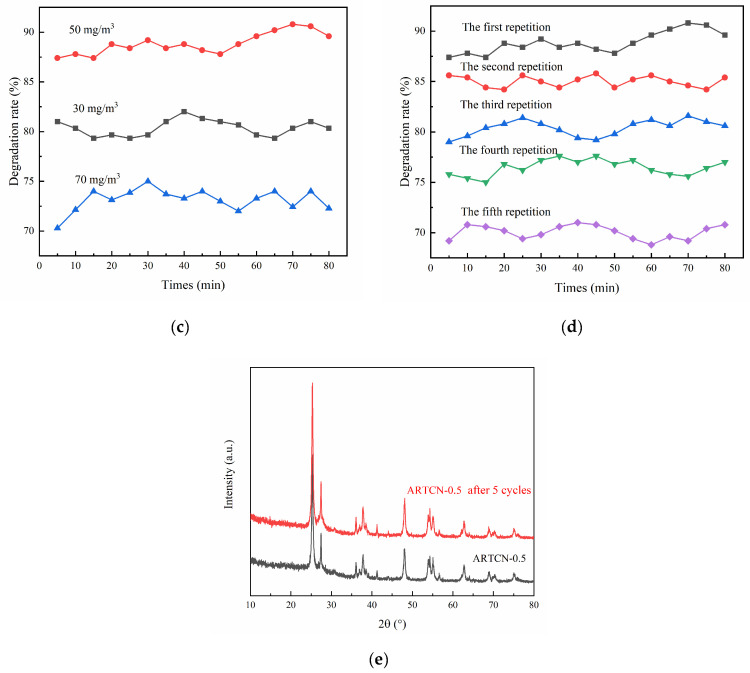
Influence of different catalysts on the degradation of gaseous ammonia (**a**), the effect of different humid air flow rates on the degradation of gaseous ammonia (**b**), the effects of different ammonia concentrations on the degradation of gaseous ammonia (**c**), the repeated degradation performance of ARTCN-0.5 (**d**), and XRD patterns before and after the repeated use (5 times) of ARTCN-0.5 (**e**).

The initial concentration of ammonia was 50 mg/m^3^, and the flow rate of the moist air was 0.9 mL/min. Different catalysts were used to study their influence on the degradation of gaseous ammonia, and the results are shown in Figure 7a. This indicated that PET itself was not good at removing gaseous ammonia, and the average degradation rates of gaseous ammonia by ART, ARTCN-0.1, ARTCN-0.5, ARTCN-1, and CN were 52.35%, 61.43%, 88.86%, 63.90%, and 33.51%, respectively. These results indicated that the degradation performance of gaseous ammonia by the ARTCN-X composite catalyst modified by CN was improved, and the effect was most obvious when the amount of CN was 0.5 g because ARTCN-0.5 had the strongest photogenerated carrier separation ability.

The initial concentration of ammonia was 50 mg/m^3^, and the flow rate of moist air was adjusted. ARTCN-0.5 was selected to study its influence on the degradation of gaseous ammonia, and the results are shown in Figure 7b. At 1.2 mL/min, the degradation rates of gaseous ammonia by ARTCN-0.5 were 57.93%, 63.98%, 78.26%, 83.38%, 88.86%, 87.10%, and 67.04%. The degradation rates of gaseous ammonia by ARTCN-0.5 first increased and then decreased with the flow rate of moist air. This was because moist air was conducive to the deposition of gaseous ammonia. However, ARTCN-0.5 could oxidize water molecules into hydroxyl radicals (·OH) with strong oxidation in the photocatalytic reaction process, thus improving the efficiency of the photocatalytic reaction. However, ammonia molecules could not tightly bind to the catalyst and flow out of the system before being reacted, resulting in a decrease in its degradation rate.

When the flow rate of moist air was 0.9 mL/min, ARTCN-0.5 was selected to study its influence on the degradation of gaseous ammonia, and the results are shown in Figure 7c. When the concentrations of gaseous ammonia were 30 mg/m^3^, 50 mg/m^3^, and 70 mg/m^3^, the average degradation rates of gaseous ammonia by ARTCN-0.5 were 80.38%, 88.86%, and 73.15%, respectively. The degradation rate decreased if the concentration of gaseous ammonia was too low or too high. This was because ARTCN-0.5 released a limited number of active free radicals after it degraded ammonia when the concentration of gaseous ammonia was too large. Many gaseous ammonia molecules went along with the airflow, and when the concentration of gaseous ammonia was too small, the active radicals could not completely combine with the gaseous ammonia.

The initial concentration of ammonia was 50 mg/m^3^, and the flow rate of moist air was 0.9 mL/min. After the degradation performance of ARTCN-0.5 was tested, PET loaded with ARTCN-0.5 was removed and kept in an oven at 70 °C for 2 h. Then, the test was repeated to explore the reuse performance of ARTCN-0.5; the results are shown in Figure 7d. The average degradation rates for the five reuse cycles were 88.86%, 85.00%, 80.40%, 76.48%, and 70.05%. Given the inevitable catalyst loss in the process of repeated testing, ARTCN-0.5 demonstrated a good overall repeatable degradation performance.

Figure 7e shows the XRD patterns of ARTCN-0.5 before and after repeated use (five times). We found that the crystal shape and structure of ARTCN-0.5 did not significantly change after five repeated uses, indicating that its properties were stable.

#### 2.2.2. Study on the Photocatalytic Degradation of Liquid Ammonia

**Figure 8:**
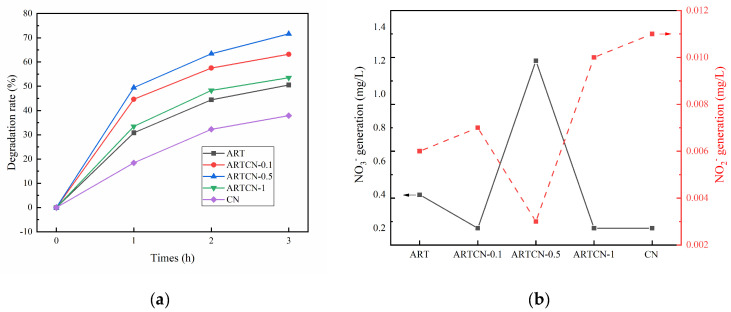
Degradation of the liquid ammonia by samples (**a**); NO_3_^−^ and NO_2_^−^ production after the reaction (**b**).

When pH > 10, NH_4_^+^ hydrolyzes in an alkaline solution and then exists in the form of NH_3_·H_2_O [39]. Therefore, we chose to adjust the pH of ammonia nitrogen to 10.1, and then we tested the concentration of ammonia nitrogen in the solution. We found that it decreased by 3.82% because a small amount of NH_3_·H_2_O escaped in the gaseous form. The degradation results of ammonia nitrogen by different catalysts are shown in Figure 8a, where the catalytic dose was 50 mg and the initial concentration of NH_4_^+^ was 100 mg/L. The degradation rates of liquid ammonia by ART, ARTCN-0.1, ARTCN-0.5, ARTCN-1, and CN after 3 h of reaction were 50.54%, 63.18%, 71.60%, 53.55%, and 37.91%, respectively, indicating that the degradation performance of the gaseous ammonia by the ARTCN-X composite catalyst after CN improvement was improved.

When the initial concentration of ammonia nitrogen was 100 mg/L, 50 mg of ARTCN-0.5 catalyst was added, the pH was 10.1, and the reaction was performed after 3 h. The results are shown in Figure 8b, which shows that the NO_3_^−^ and NO_2_^−^ concentrations were not greater than 1.4 and 0.012 mg/L, respectively. According to the literature reports, there are three main reaction products of ammonia nitrogen, nitrate, nitrite, and nitrogen, and ammonia nitrogen is mainly oxidized to N_2_ [40].

## 3. Discussion

### 3.1. Charge Transfer Mechanism Discussion

To understand the reason for the observed improved photocatalytic performance, we proposed a charge separation and transfer mechanism. The conduction band position of a semiconductor could be calculated by the empirical formulas shown in Equations (2) and (3) [41]:

Ec = χ − Ee − Eg/2(2)

Ev = Ec + Eg(3)

where χ is the geometric mean of the absolute electronegativity of each atom in the semiconductor, with TiO_2_ [42] and g-C_3_N_4_ [43] χ values of 5.81 eV and 4.82 eV, respectively; Ee is a constant relative to the standard hydrogen electrode of about 4.5 eV [44]; Eg is the semiconductor band gap width; Ev is the semiconductor valence band energy; and Ec is the semiconductor conduction band energy.

According to the analysis presented in Figure 5b, the Eg values of TiO_2_ and g-C_3_N_4_ were 3.01 and 2.84 eV, respectively. We calculated that the Ev and Ec values of ART were 2.815 and −0.195 eV, respectively, the Ev and Ec of g-C_3_N_4_ had a value of 1.59, and Ec was −1.25 V(vs NHE). CN was more negative than ART, and the Ev of ART was higher than that of CN. The high photocatalytic activity of the heterojunctions between ARTCN could be explained by the following mechanism.

**Figure 9:**
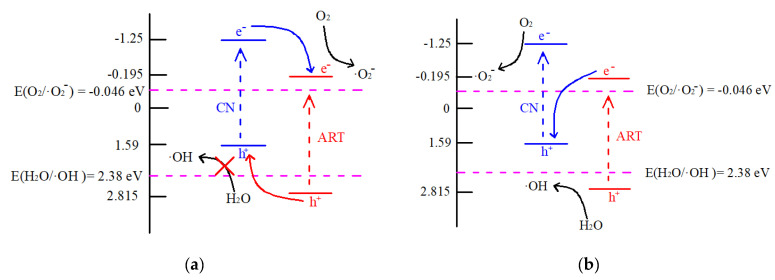
Charge transfer mechanism of the photocatalytic degradation conventional type II heterojunction (**a**) and Z-type heterostructure (**b**).

**Figure 10:**
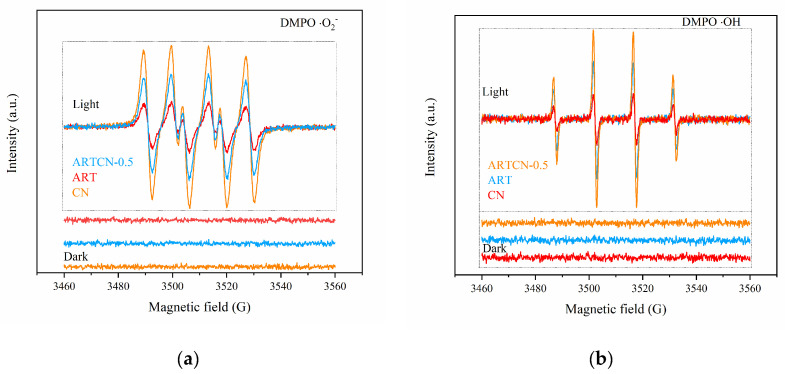
ESR profiles of DMPO ·O_2_^−^ (**a**) and DMPO ·OH (**b**) of the samples.

Two possibilities existed for the photocatalytic mechanism of the composite catalyst: (1) conventional type II heterojunctions and (2) Z-type heterojunctions [45]. Under simulated solar irradiation, ART and CN were excited and generated electron-hole pairs. If the type II heterojunction mechanism was followed, the photogenerated electrons in the conduction band of CN would be transferred to the conduction band of ART and the photogenerated holes in the valence band of ART would be transferred to the valence band of CN [46], as shown in Figure 9a. However, the hole in the valence band of CN could not generate ·OH by reacting with H_2_O. This was because the valence band potential of CN (1.59 eV) was lower than the standard oxidation potential E (H_2_O/·OH) (2.38 eV) [47], resulting in a decrease in ·OH content, which was inconsistent with the results shown in Figure 9b. If the charge transfer mechanism of the Z-type heterostructure was followed, the photogenerated electrons in the ART conduction band would transfer to the valence band of CN and combine with the photogenerated holes in the CN valence band. This would result in a reduction in electrons in ARTCN and the accumulation of electrons in the CN conduction band and holes in the ART valence band, which was why the content of ·O_2_^−^ produced by CN shown in Figure 10a was higher than that of ARTCN. The holes that accumulated in the valence band of ART had strong oxidability and could directly degrade ammonia molecules. However, the Ec (−1.10 V) of CN was more negative than E_O_ (O_2_/·O_2_^−^), and the electrons that accumulated in the conduction band of CN could react with O_2_ to generate ·O_2_^−^. This z-type heterostructure was more consistent with the characterization results of ESR (Figure 10).

**Figure 11:**
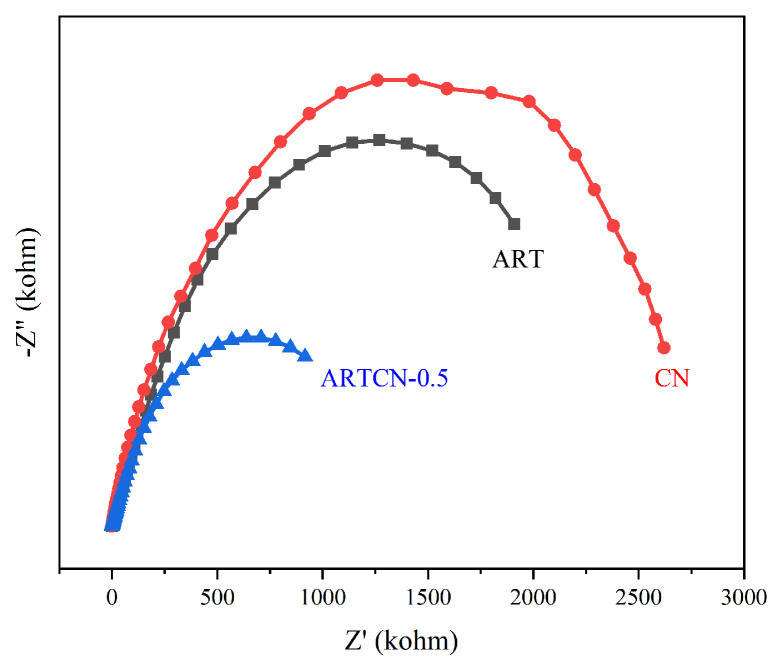
EIS spectra of the samples.

To further verify the accuracy of the above results, the test results of EIS are presented in Figure 11, which shows the impedance diagrams of ART, CN, and ARTCN. The electron transfer resistance in the sample was equivalent to the semicircle diameter on the EIS diagram, where the smaller the arc radius, the lower the charge transfer resistance of the composite sample [48]. Hence, due to the formation of the Z-type heterostructure, the charge-transfer resistance of ARTCN-0.5 was lower than ART and CN, which significantly improved the efficiency of photogenerated electron-hole migration and separation, which was consistent with the work of Y.Y. Wang [49].

### 3.2. Photocatalytic Degradation Mechanism Discussion

ESR was used to detect the types of free radicals in the catalysts under light, and then we explored their degradation mechanism of ammonia. As shown in Figure 10a,b, ART, CN, and ARTCN-0.5 did not produce free radicals in the absence of light, and superoxide free radicals (·O_2_^−^) and hydroxyl free radicals (·OH) were detected after light exposure. ·O_2_^−^ and ·OH played decisive roles in the entire reaction [50]. The degradation mechanism was as follows. After gaseous ammonia combined with moist air, NH_4_^+^ was present as NH_3_·H_2_O under alkaline conditions. First, when the catalyst was illuminated, electrons and holes were generated [51]. The holes oxidized the water molecules on the surface of the catalyst, forming ·OH, while the electrons and dissolved oxygen underwent a series of reactions to form ·O_2_^−^, following Equations (4)–(6) [52]. ·OH and·O_2_^−^ were the main active components in the photocatalytic reaction process, and they could rapidly oxidize the NH_4_^+^/NH_3_/NH_3_·H_2_O adsorbed on the surface of the catalyst [53]. Equations (9)–(13) show that ammonia was directly and completely oxidized to N_2_ [54], and the NO_3_^−^ and NO_2_^−^ contents were very low after the reaction. Equations (12)–(16) show that ammonia was not completely oxidized to NO_3_^−^ and NO_2_^−^, which was consistent with the research of Sun et al. [55].

g-C_3_N_4_/(A-R)TiO_2_+hv → g-C_3_N_4_/(A-R)TiO_2_ (h^+^ + e^−^) (4)

h^+^ + H_2_O → H^+^ + ·OH (5)

e^−^ + O_2_ →·O_2_^−^ (6)

NH_3_ + ·OH → NH_2_ + 2H_2_O (7)

NH_2_ + ·OH → NH + H_2_O (8)

NH + ·OH → N + H_2_O (9)

NH_x_ + NH_y_ → N_2_H_x+y_ (x, y = 0, 1, 2) (10)

N_2_H_x+y_ + (x+y)OH → N_2_ + (x+y) H_2_O (11)

NH_3_ + ·OH (h^+^) → NH_2_OH + H^+^ (12)

NH_2_OH + ·O_2_^−^ →·O_2_NHOH (13)

O_2_NHOH + OH → NO_2_^−^ + H_2_O + ·OH (14)

NO_2_^−^ + OH → HONO_2_ (15)

HONO_2_ → NO_3_^−^ + H^+^ (16)

## 4. Materials and Methods

### 4.1. Materials

Ammonium chloride (AR, Chengdu Colon Chemicals Co., Ltd. Chengdu, Sichuan province, China), butyl titanate (AR, Chengdu Colon Chemicals Co., Ltd. Chengdu, Sichuan province, China), absolute ethanol (AR, Chongqing Chuandong Chemical Co., Ltd. Nanan district, Chongqing, China), urea (AR, Sinopharm Chemical Reagent Co., Ltd. Huangpu district, Shanghai, China), and NaOH (AR, Chongqing Chuandong Chemical Co., Ltd. Nanan district, Chongqing, China) were used in this study.

### 4.2. Preparation of Catalysts

We added 30 mL of absolute ethanol to 35 mL of butyl titanate, which was denoted as solution A. Then, we added 30 mL of absolute ethanol to 100 mL of distilled water, which was denoted as solution B. Solution A was dropwise added to solution B at 4 drops per second, and then it was mixed and stirred at a low speed for 2 h to obtain the TiO_2_ gel, which was aged at room temperature. The aged TiO_2_ gel was transferred to a stainless steel reaction kettle with a polytetrafluoron liner and maintained at 100 °C for 2 h. After cooling, it was centrifuged and settled, washed with deionized water and ethanol 3 times, and dried in a 100 °C air-drying oven. Then, the TiO_2_ catalyst powder was obtained after grinding. The TiO_2_ powder was maintained at 600 °C for 2 h at a heating rate of 10 °C/min, and then it was cooled to room temperature and ground to obtain ART.

Subsequently, 10 g of urea and 50 mL of water were added to the crucible and evenly stirred. The crucible was placed in a muffle furnace and maintained at 500 °C for 2 h at a heating rate of 10 °C/min. After the heating program was finished and the muffle furnace naturally cooled, the obtained bulk particles were ground to obtain CN.

We mixed 0.1 g of CN, 0.5 g of CN, 1 g of CN, and 1 g of ART; added 5 mL of absolute ethanol; stirred evenly; and then separated and dispersed the mixture using a cell fragmentation apparatus (Ningbo, Zhejiang Province, China. Xinzhi Biotechnology Co., Ltd., SCIENTZ-IID, 65 Hibiscus Road, Ningbo National High-tech Zone). Maintaining the temperature at 300 °C for 2 h at a heating rate of 10 °C/min, ARTCN-X was obtained and is denoted as ARTCN-0.1, ARTCN-0.5, and ARTCN-1.

### 4.3. Fixation of Photocatalytic Materials

**Figure 12:**
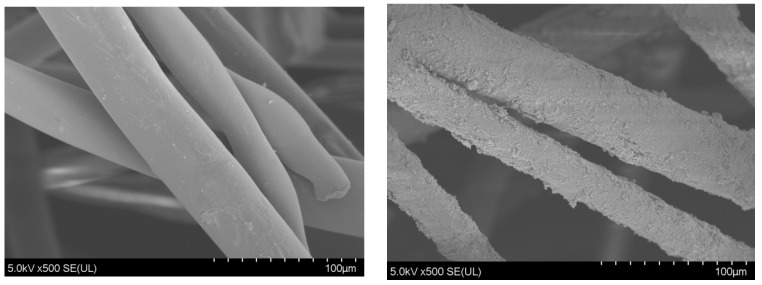
SEM of the PET scan before and after catalyst support.

We washed the polyester fiber cotton (PET) with 1 mol/L of NaOH to remove the surface impurities, and then we dried and set it aside. Subsequently, 100 mg of catalyst was dissolved in water, the treated PET was added and shaken in a shaker for 30 min, and the water on the surface and the excess catalyst were drained before the catalyst was dried at 70 °C for later use. The PET scanning electron microscope results before and after catalyst loading are shown in Figure 12. We clearly observed that the photocatalytic materials werare evenly loaded on PET.

### 4.4. Catalyst Characterization

A D8 Advance model X-ray diffractometer (XRD, Bruker, Germany) was used to analyze the crystal characteristics of the catalyst. The operating parameters were a Cu X-ray tube target and a scanning range of 10–80°. An HR800 laser confocal Raman spectrometer (Raman, Horiba Jobin Yvon, France) was used to detect the sample structures, where the excitation wavelength was 633 nm. A TriStar II 3020 series automatic specific surface analyzer (BET, GA, USA) was used to determine the specific surface area and porosity of the catalyst. The catalyst was pretreated under vacuum degassing at 200 °C for 5 h, and high-purity nitrogen was used as the adsorbent at 77 K. Then, a UV–Vis diffuse reflectance (UV–Vis DRS, Hitachi, Japan) instrument (model U-3010) was used to test the optical properties of the catalyst in the range of 200–800 nm. An F-2700 fluorescence spectrophotometer (PL, Japan, Hitachi) was used to measure the electron hole recombination, with an operating voltage of 250 V, a wavelength of 5 nm, and an excitation wavelength of 300 nm. The surface morphology and elemental distribution of the samples were analyzed by scanning electron microscopy (SEM, Sigma500, Germany) and an energy dispersive spectrometer (EDS, Bruker, Germany). Transmission electron microscopy (TEM, FEI TalOS F200S, USA) was used to analyze the lattice spacings of the samples. Fluorescence spectrophotometry (PL, HORIBA, Osaka, Japan) was used to test the photogenerated carrier separation of the catalyst with a hydrogen light source with a pulse width of 1.0 to 1.6 ns, which was used to test the fluorescence lifetime of the sample. Electron paramagnetic resonance (EPR, Bruke, Germany) was performed at room temperature using an A300 spectrometer, and 5,5-dimethyl-1-pyrrolidine N-oxide (DMPO) was used as the spin capture agent for ESR analysis. A CHI-660C electrochemical workstation was used for transient photocurrent measurements. A Na_2_SO_4_ aqueous solution (1 M) was used as the electrolyte solution, and electrochemical impedance spectroscopy (EIS, Chenhua, China) measurements were performed under visible light irradiation with frequencies ranging from 4 × 10^6^ to 1 × 10^−2^ Hz.

### 4.5. Photocatalytic Activity Tests

#### 4.5.1. Degradation of Gaseous Ammonia

**Figure 13:**
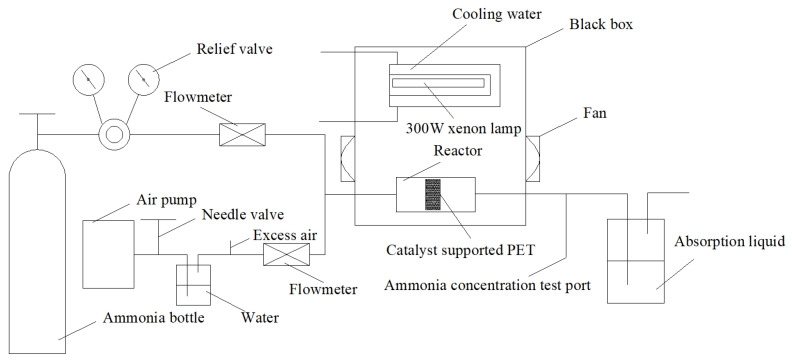
Device diagram of the photocatalytic degradation of gaseous ammonia.

**Figure 14:**
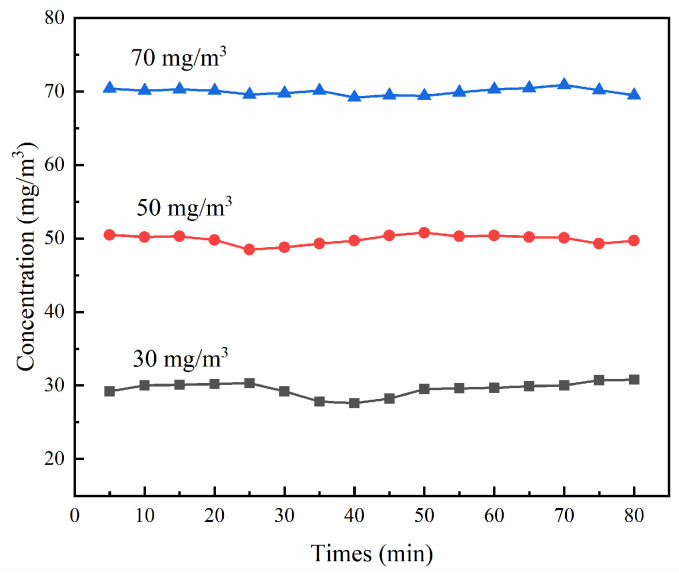
System stability test.

Figure 13 shows the device diagram for the photocatalytic degradation of gaseous ammonia. The light source consisted of a 300 W xenon lamp (30.2 mW/cm^2^), which was installed in a quartz water pipe with a condensation cycle to absorb the heat generated by illumination. Just below the xenon lamp was a photocatalytic quartz reaction tube, and PET loaded with the photocatalytic materials was placed in the tube. After exiting the cylinder, ammonia entered the reaction tube through a flowmeter. The different concentrations of standard ammonia (mixed with nitrogen) were 30 mg/m^3^, 50 mg/m^3^, and 70 mg/m^3^, with a flow rate of 100 mL/min, and the stability test results of the different concentrations of ammonia are shown in Figure 14. Air entered the photocatalytic reaction tube through the water, needle valve, and flowmeter in turn (moist air was the only source of oxygen). Ammonia was mixed with air at a certain humidity to simulate gaseous ammonia. After connecting each pipeline, the standard gas was ventilated to determine whether there was air leakage. The system was run for 10 min until it was stable, and the degradation efficiency of the gaseous ammonia in the entire process was η_1_ (Equation (17)):

η_1_ = (C_01_ − C_1_) × 100%/C_01_ (17)

where C_01_ is the standard concentration of gaseous ammonia (mg/m^3^) and C_1_ is the concentration of gaseous ammonia in the reaction process (mg/m^3^).

#### 4.5.2. Degradation of Liquid Ammonia

The photoreactor consisted of a 200 mL double-layer quartz beaker, for which the outer layer was permeated with cooling water to ensure a constant reaction temperature and the inner layer consisted of 100 mg/L of an ammonia nitrogen solution. The pH was adjusted to 10.1 by sodium hydroxide. The 300 W xenon lamp (30.2 mW/m^2^) was used as the light source to simulate sunlight while ensuring that the distance between the xenon lamp and the liquid level was 15 cm. Then, 50 mg (0.5 g/L) of catalyst was added to the solution and stirred at medium speed at room temperature; 2 mL of the sample from the reaction solution was extracted every 60 min and centrifuged for 10 min with a 10,000 r/min high-speed centrifuge, and the supernatant was obtained to determine the concentration of ammonia nitrogen and calculate its degradation rate η_2_ (Equation (18)). The nitrate and nitrite concentrations were determined after the reaction:

η_2_ = (C_02_ − C_2_) × 100%/C_02_ (18)

where C_02_ is the initial concentration of ammonia nitrogen and C_2_ is the concentration of ammonia nitrogen in the reaction process (mg/L).

## 5. Conclusions

A g-C_3_N_4_/(A-R)TiO_2_ composite catalyst (ARTCN) was prepared by using g-C_3_N_4_ (CN) as the amendment, and the amount of CN had a great influence on the performance of ARTCN. When the amount of CN was 0.5 g, ARTCN-0.5 had a better dispersion, a smaller band gap width, a larger specific surface area, a stronger light absorption capacity, and a stronger photogenerated carrier separation ability than ART.

The air wetness and initial concentration of the ammonia had a great influence on the degradation of the gaseous ammonia. When the initial concentration of ammonia was 50 mg/m^3^ and the flow rate of the moist air was 0.9 mL/min, the degradation rate of gaseous ammonia by ARTCN-0.5 reached 88.86%, and it had good repeatability. When the catalytic dose was 50 mg and the initial concentration of NH_4_^+^ was 100 mg/L, the degradation rate of the liquid ammonia by ARTCN-0.5 was 71.60% at 3 h, and small amounts of NO_3_^−^ and NO_2_^−^ were generated. Subsequently, ·OH and ·O_2_^−^ were the main active components in the photocatalytic reaction process.

The photogenerated electrons in the conduction band of ART transferred to the valence band of CN and combined with the photogenerated holes in the valence band of CN, forming a Z-type heterostructure that significantly improved the efficiency of the photogenerated electron-hole migration and separation, thus increasing the reaction rate.

## Figures and Tables

**Table 1:** Rutile content of the catalysts.

Samples	I_R_ (2θ = 27.24°)	I_A_ (2θ = 25.28°)	X_R_
ART	523.48	2131.93	23.49%
ARTCN-0.1	605.19	2260.33	25.06%
ARTCN-0.5	704.02	2110.14	29.43%
ARTCN-1	953.81	2251.77	34.61%

**Table 2:** Structural parameters of the samples.

Samples	Specific Surface Area (m^2^/g)	Pore Volume (cm^3^/g)	Pore Diameter (nm)
ART	27.28	0.08	11.28
ARTCN-0.1	33.92	0.09	10.13
ARTCN-0.5	36.16	0.08	9.65
ARTCN-1	38.08	0.12	9.29
CN	42.09	0.11	8.67

## Data Availability

Not applicable.
